# A review of biomarkers in peri-miniscrew implant crevicular fluid (PMICF)

**DOI:** 10.1186/s40510-017-0195-8

**Published:** 2017-11-27

**Authors:** Avinash Kaur, Om P. Kharbanda, Priyanka Kapoor, Dinesh Kalyanasundaram

**Affiliations:** 1 Centre for Biomedical Engineering, Indian Institute of Technology Delhi, New Delhi, India; 2 Centre for Dental Education and Research, All India Institute of Medical Sciences, New Delhi, India; 3 Department of Orthodontics, Faculty of Dentistry, Jamia Millia Islamia, New Delhi, India

**Keywords:** Biomarker, Peri-miniscrew implant crevicular fluid (PMICF), Orthodontic tooth movement (OTM), IL-1β, IL-2, IL-6, IL-8, TNF-α, RANKL, OPG

## Abstract

**Background:**

The temporary anchorage devices (TADs) which include miniscrew implants (MSIs) have evolved as useful armamentarium in the management of severe malocclusions and assist in complex tooth movements. Although a multitude of factors is responsible for the primary and secondary stability of miniscrew implants, contemporary research highlights the importance of biological interface of MSI with bone and soft tissue in augmenting the success of implants.

The inflammation and remodeling associated with MSI insertion or loading are reflected through biomarkers in peri-miniscrew implant crevicular fluid (PMICF) which is analogous to the gingival crevicular fluid. Analysis of biomarkers in PMICF provides indicators of inflammation at the implant site, osteoclast differentiation and activation, bone resorption activity and bone turnover. The PMICF for assessment of these biomarkers can be collected non-invasively via paper strips, periopaper or micro capillary pipettes and analysed by enzyme-linked immunosorbent assay (ELISA) or immunoassays. The markers and mediators of inflammation have been previously studied in relation to orthodontic tooth movement include interleukins (IL-1β, IL-2, IL-6 and IL-8), growth factors and other proteins like tumour necrosis factor (TNF-α), receptor activator of nuclear factor kappa-B ligand (RANKL), chondroitin sulphate (CS) and osteoprotegerin (OPG).

Studies have indicated that successful and failed MSIs have different concentrations of biomarkers in PMICF. However, there is a lack of comprehensive information on this aspect of MSIs. Therefore, a detailed review was conducted on the subject.

**Results:**

A literature search revealed six relevant studies: two on IL-1β; one on IL-2, IL-6 and IL-8; one on TNF-α; one on CS; and one on RANKL/OPG ratio. One study showed an increase in IL-1β levels upon MSI loading, peak in 24 hours (h), followed by a decrease in 21 days to reach baseline in 300 days. A 6.87% decrease in IL-2 levels was seen before loading and a 5.97% increase post-loading. IL-8 showed a 6.31% increase after loading and IL-6 increased by 3.08% before MSI loading and 15.06% after loading. RANKL/OPG ratio increased in loaded compared to unloaded MSIs.

**Conclusions:**

Cytokines (mainly ILs and TNF-α) and RANKL/OPG ratio showed alteration in PMICF levels upon loading of MSIs as direct or indirect anchorage.

## Background

**Fig. 1:**
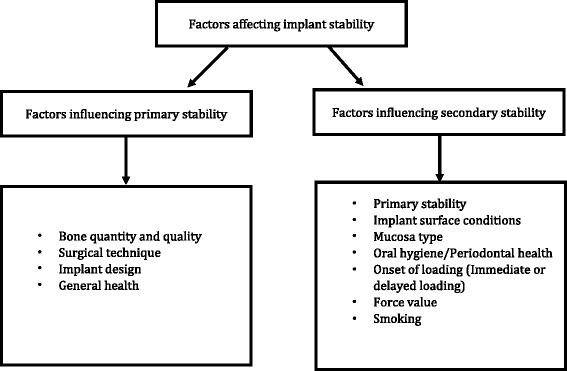
Factors affecting implant stability

Miniscrew implants (MSIs) also known as temporary anchorage devices (TADs) are used for enhancing anchorage and are now a well-accepted armamentarium in clinical orthodontic practice. The gaining popularity of MSIs in varied orthodontic settings is due to their ease of placement and removal, multitude of applications as direct or indirect anchorage, affordable cost as well as minimal surgical procedures required in their placement. The MSIs can have immediate or delayed loading protocols [1]. The MSIs success reported to have an average survival rate of 84% (range 57–95.3%) which is largely dependent on factors governing primary and secondary stability [2]. The primary stability pertains to the mechanical holding of MSI in the bone [3], while the secondary stability relates to biological retention [4]. Broadly, the factors affecting the stability can be grouped into a host, miniscrew implant and technique-related factors (Fig. 1). Contemporary research has focused on the biological seal between the implant and host tissue, more so on its surrounding peri-implant (PI) oral epithelium.

The peri-miniscrew implant crevicular fluid (PMICF), an inflammatory exudate secreted in the crevice between MSI and PI tissue, has been investigated to monitor the levels of biomarkers at multiple observation times upon MSI insertion as well as loading [5]. The composition of PMICF is analogous to other body fluids as gingival crevicular fluid (GCF) and includes inflammatory biomarkers. These are interleukins (ILs) (IL-1β, IL-2, IL-6, IL-8), growth factors and other proteins such as tumor necrosis factor (TNF)-α, receptor activator of nuclear factor kappa-B ligand (RANKL), chondroitin sulphate (CS) and osteoprotegerin (OPG). The contents of PMICF are analogous to the ones released in orthodontic tooth movement (OTM) in the GCF. The biomarkers of peri-implantitis seen in PMICF are also similar to those released in GCF during periodontitis and gingivitis [6].

Information on biomarkers in PMICF and their association with the success of MSI, though available, remains scattered. Hence, this review aims to study the biomarkers in PMICF associated with insertion of MSI, different loading practices and their impact on the stability of MSIs in the bone to finally deduce the evidence mechanism of success and failure related to inflammatory biomarkers present in PMICF.

### Host response to MSI insertion in the bone

An immediate host response to MSI insertion in the bone begins with a clot formation where cellular migration occurs comprising of osteoprogenitor cells, angiogenesis and protein surge including osteopontin, bone sialoproteins and glycosaminoglycans [7].

At a microscopic level, a hypothesis of micro-crack propagation in the bone upon MSI insertion has been proposed, probably due to elasticity difference between the bone and MSI structure [7]. A study done on beagle dogs quantified this damage as a fractional micro-cracked area, fractional diffuse damage and the total fractional injured area in adjacent (0–0.5 mm) and distant (0.5–1 mm) region. The percentage of damage was found to vary with the type of implants, self-drilling or non-drilling and thickness of the bone [2].

The repair of these micro-cracks is believed to occur by a micro-callus formation triggered by calcium phosphate leading to the creation of mineralized bone. However, in the case of any inflammation or microbial invasion, if acidification occurs, this process may get delayed [8]. Additionally, bone necrosis has also been suggested due to heat generation at a critical temperature of 47 °C for 1 min on frictional contact of bone with MSI [9, 10]. In temperatures above this, the secondary stability of MSIs gets compromised due to denaturation of proteins leading to cell death. Understanding the host factors at PI bony interface is thus imperative to ensure the secondary stability of implants, for which the biomarkers released in PMICF play a very significant role.

### Understanding MSI-soft tissue interface

Comprehension of the biological seal in the transmucosal region of MSI soft tissue interface is integral to understanding the unexplained variables governing the success of miniscrew implants. The histology of PI tissue comprises of keratinized stratified squamous oral epithelium and non-keratinized sulcular epithelium with cellular arrangement favourable for leakage of neutrophils from sub-epithelial connective tissue and mediators, in cases of peri-implantitis or inflammation. Hence, the integrity of this region is precarious for short- and long-term success of MSIs which may be corroborated based on the biochemical milieu of PMICF that shows a variation in levels of mediators in the presence of peri-implant inflammation [11].

## Results

### Biomarkers in PMICF: markers of MSI success

PMICF is an inflammatory exudate that surrounds the MSI crevice with a composition similar to the GCF, comprising of inflammatory biomarkers (such as IL-1β and IL-2, IL-6, IL-8), growth factors and other proteins (TNF-α, RANKL, CS and OPG) [11]. During inflammation, the amount of peri-miniscrew crevicular fluid as well as the concentration of biomarkers in the fluid increases. Hence, to understand the underlying biological processes, it is imperative to analyze the mediators of inflammation at multiple observation times. The non-invasive collection methods via paper strips, periopaper or micro-capillary pipettes are the standard methods of PMICF collection [5]. A literature search has identified the following mediators of interest in PMICF:

I. Cytokines

Cytokines are produced by various types of cells such as macrophages, B lymphocyte, T lymphocyte, mast cells, endothelial cells, fibroblast and stromal cells. The cytokines are significant in the modulation of inflammation through the process of cell signaling via autocrine, paracrine and endocrine signaling. They regulate the cell maturation, growth and responsiveness of specific cell population and include chemokines, interferons, ILs, lymphokines and TNFs.

1. Interleukins

ILs are a group of pro-inflammatory cytokines produced by fibroblast, osteoclast and polymorphonuclear leukocytes (PMNLs) and are responsible for bone turnover and remodeling process [12]. They include several markers such as IL-1β, IL-2, IL-6 and IL-8, that can be detected in crevicular fluids of the oral cavity including GCF and PMICF.

1.1 Interleukin-1β

IL-1β is a cytokine which is produced by the IL1B gene [13]. It is a member of the interleukin-1 family of cytokines and an essential mediator of the inflammatory response. It plays an important role in various types of cellular activities, such as differentiation, cell proliferation and apoptosis, with a potential role in bone metabolism, bone resorption and inhibition of bone formation. It works through a synergistic activity with TNFs in osteoclast differentiation by RANK-RANKL binding on the osteoblast surface [13].

IL-1β is produced in a biologically inactive form that requires protease-mediated cleavage to govern its proinflammatory functions. Inflammasome complex is formed during caspase-1-mediated cleavage of IL-1β that is responsible for secretion of bioactive IL-1β in many disease models [14]. Its levels have been reported to be very high in inflamed gingival tissues [15] and also in the crevicular fluid of diseased implant sites when compared to healthy implant sites [16].

A study by Sari et al [17] assessed levels of IL-1β  in PMICF surrounding 20 implants used as direct anchorage in MSI group and compared it to levels of IL-1β in GCF of treatment group (maxillary canines). PMICF in this study was collected at 1 hour(h), 24 h, 48 h, 168 h, 14 days and 21 days after loading. The IL-1β levels were higher in the treatment group at 24 h (37.8 ± 6.7 pg/μL) than those in the implant (22.0 ± 2.5 pg/μL) and control (19.6 ± 2.6 pg/μL) groups. No significant change was observed between the control and implant groups for all the above periods [17]. Thus, this study favored the use of implants for absolute anchorage.

Another study by Monga et al. [5] evaluated levels of IL-1β in PMICF of MSIs used as an indirect anchorage in 11 patients with all first premolar extractions. The MSIs were loaded after a delay of 3 weeks using 200-g Nitinol closed coil springs of 9-mm length for en masse retraction. PMICF was collected at nine different time intervals and the levels were significantly higher at 1 h after MSI placement (0.27 pg/μL) and 1 day after loading (0.27 pg/μL) as compared to the baseline (0.13 pg/μL). However, the levels decreased after 21 days (0.15 pg/μL) and 72 h after loading (0.14 pg/μL). The decline in the levels of IL-1β around miniscrew 21 days after loading towards the baseline is suggestive of an adaptive bone response to stimulus and consequent cessation of active inflammation [5].

1.2 Interleukins 2, 6 and 8

Other proinflammatory cytokines, IL-2, IL-6 and IL-8, are also proven markers for periodontal (pdl) inflammation and alveolar bone resorption during OTM [18] and are thus potential biomarkers for inflammation in MSI insertion and loading. Of these, IL-2 produced by T helper 1 cells stimulates macrophages, natural killer cells and T cell proliferation, which in turn activate cellular immune response [19]. It also encourages osteoclast activity during bone resorption and also plays a vital role in the pathogenesis of the periodontal disease [20]. IL-6, on the other hand, is involved in differentiation of CD4 T cells [21, 22] and induction of osteoclastic bone resorption by mediating osteoclastogenesis [23]. The presence of IL-6 in human gingival tissues and cells indicates its involvement in molecular events associated with inflammatory periodontal  diseases [23]. There is ample evidence of high levels of IL-1β and IL-6 in inflamed gingival tissues when compared to uninflamed tissues in young adults, thus potentiating its role in inflammation. Apart from these, IL-8 is a chemokine produced by macrophage, epithelial cells and endothelial cells and is essential in early inflammatory response [18] with a role in neutrophil recruitment and degranulation during inflammation [24]. Evidence also supports high IL-8 levels in periodontitis and at PI inflammation sites [25]. A study by Tuncer et al. [26] revealed high levels of IL-8 at pdl tension sites during canine retraction that served as a triggering factor for bone remodeling.

Hamamci et al. [27] evaluated levels of IL-2, IL-6 and IL-8 in GCF and PMICF of 16 patients undergoing en masse retraction of anterior teeth using MSIs as direct anchorage. Samples were collected from GCF of treatment teeth (maxillary canines), control teeth (second premolars) and PMICF surrounding MSIs in implant group. The sample collection began at 2 weeks after MSI insertion (baseline) followed by six observation time points. Results showed levels of IL-2 (74.19 ± 30.36 pg/μL) and IL-8 (83.52 ± 14.34 pg/μL) were higher in implant group than in control group at 24 h after loading, while IL-6 levels remained unchanged at all observation time points in all three groups. These observations suggest that the force applied to miniscrews during orthodontic loading may lead to cytokine secretion and cause screw loosening.

1.3 Tumour necrosis factor (TNF-α)

There is a proven role of TNF-α, a pro-inflammatory cytokine, in regulating and amplifying the inflammatory response in periodontal and peri-implant tissues [28]. It is produced by monocytes, macrophages, and osteoblasts and stimulates fibroblast cells to produce collagenase. Lowney et al. [29] reported an increase in TNF-α in GCF upon application of orthodontic force. Another study could not find a significant increase in levels of TNF-α in GCF/PMICF after 21 days of de novo plaque accumulation [30].

Kaya et al. [28] estimated TNF-α levels around MSIs during canine distalisation by collecting GCF and PMICF samples of treatment (maxillary canines), miniscrew (implant) and control (maxillary second premolars) group. The samples were collected before and after loading at 1, 24, and 48 h, 7 and 21 days and 3 months. The difference in TNF-α levels was insignificant in the implant (32.00 ± 4.50 pg/μL) and control group (31.91 ± 7.26 pg/μL) 24 h after loading but higher in treatment group (34.75 ± 6.93 pg/μL), thus favoring MSIs for absolute anchorage.

2. Other inflammatory markers

2.1 Receptor activator of nuclear factor kappa-B ligand and osteoprotegerin

RANK/RANKL/OPG ratio is known to determine osteoclast genesis by virtue of the inter-relationships in their mechanisms of action. Of these, RANKL is a member of TNF cytokine family [31] and produced in the plasma membrane of osteoblasts and stromal cells. It is a ligand of OPG/osteoclastogenesis inhibitory factor (OCIF) and induces osteoclast differentiation and stimulates bone resorption activity. OPG, on the other hand, is a decoy receptor of RANKL produced by osteoblastic cells, which causes osteoclast apoptosis [32]. The biological impact of OPG on bone cells incorporates inhibition of terminal phases of osteoclast differentiation, suppression of activated matrix osteoclasts and induction of apoptosis. Hence, bone remodeling is regulated by a balance between RANK-RANKL binding and OPG production [33] which has also been assessed in PMICF. A study by Enhos et al. [34] measured levels of RANKL and OPG in PMICF pre-loading and at 1, 2, 7 and 30 days after loading. A variation in OPG levels among loaded (72.74 pg/μL) and unloaded (81.06 pg/μL) groups were observed at 24 h. On the other hand, RANKL level at 24 h was higher in the loaded group (4631.25 pg/μL) than in unloaded group (3935.42 pg/μL), thus indicating more osteoclastic resorption in loaded than in the unloaded group.

2.2 Chondroitin sulphate

CS is a sulphated glycosaminoglycan with a significant role in bone and tissue destruction. It is a fundamental part of connective tissue extracellular matrix including the hyaline cartilage, contributing to its elasticity and other functions [35]. The level of CS in human GCF has been used to study alveolar bone remodeling as a result of pdl disease and orthodontic tooth movement [36]. A few investigations have observed CS in PI tissue to access the stability of dental implants and found that the levels of CS in PICF might be a successful strategy for monitoring changes in bone metabolic activity [37–40].

Intachai et al. [41] evaluated CS during unloaded (1, 3, 5 and 7 days) and loaded (14, 21, 28 and 35 days) periods from ten patients. During pre-loading, high levels of CS were found at day 1 (758.03 pg/μL) while post loading observations depicted higher levels, at 28 days (1025.11 pg/μL), but these differences were not statistically significant. Thus, implying orthodontic force on MSI does not significantly affect CS levels in PMICF.

## Discussion

The current review has attempted to generate evidence on biomarkers in PMICF in orthodontic patients (Table 1). We located a total of six studies on biomarkers present in PMICF. These included two on IL-1β, one on IL-2, IL-6 and IL-8, one on TNF-α, one on CS and one on RANKL/OPG ratio [5, 17, 27, 28, 34, 40]. The maximum duration of the study conducted for IL-1β was 300 days; IL-2, IL-6, IL-8 and TNF-α were 90 days; RANKL and OPG was 30 days; and CS was 35 days. The PMICF samples in all studies were collected using paper strips except that for IL-1β which was collected using micro-capillary pipettes [5]. Oral hygiene of patients was maintained in all the studies. Chlorhexidine gluconate (0.2%) was used as a mouthwash to reduce inflammation for the patients under orthodontic treatment only by Monga et al [5].

**Table 1:** Studies on Biomarkers in PMICF

S.No	Reference	Biomarkers	Method of collection	Analysis method	No. of patients	Duration of study (days)	Time at which significant variation was achieved	Concentration*			Percentage difference (%)
								Before loading implant	After loading implant	Control	
1	Monga et al. [5]	Interleukin-1β (IL-1β)	Micro capillary pipettes	ELISA	11	300	After 24 h of loading	0.13 pg/μL	0.27 pg/μL	-	
2	Sari et al. [17]	Interleukin-1β (IL-1β)	Paper strips	ELISA	10	21	After 24 h of loading	-	22.0 ± 2.5 pg/μL	19.6 ± 2.6 pg/μL	12.24
3	Hamamci et al. [27]	Interleukin 2 (IL-2)	Paper strips	ELISA	16	90	After 24 h of loading (Unchanged)	65.20 ± 20.73 pg/μL	74.19 ± 30.36 pg/μL	70.01 ± 31.24 pg/μL	-6.87, 5.97
4	Hamamci et al. [27]	Interleukin 6 (IL-6)	Paper strips	ELISA	16	90	After 24 h of loading (Unchanged)	3.01 ± 0.82 pg/μL	3.36 ± 0.97 pg/μL	2.92 ± 1.15 pg/μL	3.08, 15.06
5	Hamamci et al. [27]	Interleukin 8 (IL-8)	Paper strips	ELISA	16	90	After 24 h of loading	77.84 ± 12.59 pg/μL	83.52 ± 14.34 pg/μL	78.56 ± 17.10 pg/μL	-0.91, 6.31
6	Kaya et al. [28]	Tumor necrosis factor alpha (TNF-α)	Paper strips	ELISA	16	90	After 24 h of loading	31.38 ± 5.99 pg/μL	32.00 ± 4.50 pg/μL	31.91 ± 7.26 pg/μL	-1.66, 0.28
7	Enhos et al. [34]	Receptor activator of nuclear factor kappa-B ligand (RANKL)	Paper strips	ELISA	20	30	After 24 h of loading	3935.42 pg/μL	4631.25 pg/μL	-	
8	Enhos et al. [34]	Osteoprotegerin (OPG)	Paper strips	ELISA	20	30	After 24 h of loading	81.06 pg/μL	72.74 pg/μL	-	
9	Intachai et al. [40]	Chondroitin sulphate (CS)	Paper strips	ELISA	10	35	After 28 days of loading	758.03 pg/μL	1025.11 pg/μL	-	

* The concentration of biomarkers were converted into pg/µL for uniformity

Direct loading was used in all studies [17, 27, 28, 34, 40] except in a study assessing IL-1β levels which used indirect loading [5]. Levels of IL-2, IL-6 and IL-8, TNF-α, RANKL and OPG were analysed by applying a force of 150 g [27, 28, 34], while 50 g of force was applied to study the levels of CS [40]. IL-1β levels were evaluated by application of different force levels with 200g force used in a study by Monga et al. [5] and 120 g force applied in study by Sari et al [17].

The most widely considered biomarkers in PMICF are cytokines comprising IL-1β, IL-2, IL-6 and IL-8 and TNF-α, which is similar to biomarkers studied in GCF during OTM [6]. In the present review, a rise in IL-1β levels is seen immediately at MSI placement and 24 h after loading of MSIs, initially due to the trauma of insertion and later upon application of orthodontic forces [5]. A similar trend in levels of IL-1β at 24 h after activation of the orthodontic appliance has also been noticed in GCF of treatment teeth in multiple studies [6]. IL-1β peak in GCF can be explained by the release of IL-1β in the paracrine area within 1–2 h of the mechanical stimulus, further triggering the release of histamine from mast cells, and increased vascular permeability, finally leading to a peak in IL-1β levels between 24 and 48 h [42]. De novo synthesis of IL-1β along with IL-6 mRNA on application of forces for OTM has also been documented by Alhashimi et al. in the maxillary first molars of 12 rats where the maximum expression was seen in 3 days [43].This further strengthens the role of proinflammatory cytokines in remodeling of bone consequent to orthodontic force application.

Monga et al. [5] reported IL-1β levels peak in PMICF at 24 h on miniscrew insertion and 24 h after loading. The levels then gradually decrease at 21 days to reach baseline in 300 days. This may be due to inherent feedback mechanisms by the synergistic or antagonistic action of various cytokines leading to fall in levels and cessation of inflammation and restoration of pdl architecture [44].

Another study by Sari et al. [17] however showed no significant increase in IL-1β in PMICF upon loading when compared to levels in GCF of control teeth. The IL-1β levels were 92.8% higher in GCF of treatment teeth when compared to levels in PMICF of implant group as well as GCF of control teeth. Similar results have also been observed in levels of TNF-α where a significant increase was seen only in GCF of treatment teeth (maxillary canines) at 24 h when compared to control and implant groups. This implies insignificant bony resorption around MSIs when used as a direct anchorage, and hence, it supports TADs as absolute anchorage devices.

The percentage change in levels of ILs broadly showed an increase upon loading of MSIs. IL-2 in PMICF depicted 6.87% decrease in levels before MSI loading and 5.97% increase after loading. While IL-8 was 6.31% higher after loading, IL-6 was 3.08% more before MSI loading and 15.06% after loading. Nevertheless, the percentage difference was not comparable enough (IL-2, IL-6, IL-8 and IL-1β); hence, more studies need to be performed to measure the levels of IL-2, IL-6, IL-8 and IL-1β.

Studies on other cytokines receptors, RANKL and its decoy receptor OPG revealed that in PMICF of loaded implant group, the concentration of RANKL was higher than in the unloaded group while OPG/RANKL ratio was significantly reduced. This finds support in animal and in vitro studies on pdl cells where RANKL upregulation have been documented in compressive orthodontic force while OPG has been shown to inhibit RANK/RANKL interaction and thus inhibit bone resorption [45, 46].

Chondroitin sulphate is another biomarker that has been investigated in bone remodeling consequent to OTM, pdl disease and in implant stability [38, 46]. Interestingly, the results of a single study evaluating CS in PMICF have shown increased levels of CS 14 days before failure of two MSIs out of 20 MSIs placed in the subjects [40].This indicates higher bone resorption associated with a rise in levels of CS (WF6 isotope) and hence MSI mobility and failure.

### Scope of future research

There is a scope of future research at a biochemical level to evaluate all mediators associated with inflammation and bone resorption. Extracellular high mobility box protein (HMGB1) would be interesting to study for its role in the production of cytokines IL-1β, IL-6, IL-17 and RANKL. Other potent mediators would be matrix metalloproteinases (MMPs) that are zinc-dependent endopeptidases released consequent to extracellular matrix degradation and tissue remodeling. Few MMPs, namely, MMP1, 2, 3, 7, 8, 9, 12 and 13 and MMP9/NGAL, have been studied in OTM and may prove to be potential biomarkers for remodelling associated with MSI stability as well.

Besides these, circulating nucleic acids, T cells, hematopoietic cells, pentraxin-3 and macrophage colony-stimulating factor (M-CSF) play a role in osteoclast differentiation. These are few of the mediators to be explored as prognostic indicators for implant stability. The biofilm around MSIs can also contribute to inflammation and presence of inflammatory biomarkers in PMICF. The concentration of these biomarkers in PMICF, besides being influenced by MSI insertion and loading, can also vary in pdl inflammation or inflammation in soft tissue. These factors should be considered in future studies.

## Conclusions

This review provides substantiation of bone and tissue remodeling process around MSIs with or without loading. The following conclusions are drawn:

- Alteration in levels of IL-1β, IL-2, IL-6 and IL-8, TNF-α, and CS, as well as RANKL/OPG ratio was seen in PMICF on placement as well as on loading of MSIs.
- IL-1β increased in PMICF upon MSI loading, reaching a peak in 24 h, then decreased in 21 days.
- Percentage change in levels of ILs in PMICF depicted a 6.87% decrease in IL-2 levels before loading and a 5.97% increase post-loading. IL-8 showed a 6.31% increase after loading, and IL-6 increased by 3.08% before MSI loading and 15.06% after loading.
- TNF-α and CS did not show a significant variation in placement and loading of MSIs.
- RANKL/OPG ratio was higher in loaded MSIs than unloaded.

Further studies need to be conducted with robust study design to resolve heterogeneity in the current literature. In future studies, besides evaluating other biomarkers, the sample size too needs to be increased with age and sex consideration. Also, adequate observation intervals pre- and post-loading may prove to be more specific in understanding the biological factors of implant stability.

## Abbreviations

CS: Chondroitin sulphate
ELISA: Enzyme-linked immunosorbent assay
GCF: Gingival crevicular fluid
HMGB1: High mobility box protein
IL-1β: Interleukin-1β
IL-2: Interleukin-2
IL-6: Interleukin-6
IL-8: Interleukin-8
M-CSF: Macrophage colony-stimulating factor
MMPs: Matrix metalloproteinases
MSIs: Miniscrew implants
OPG: Osteoprotegerin
OTM: Orthodontic tooth movement
pdl: Periodontal
PI: Peri-implant
PMICF: Peri-miniscrew implant crevicular fluid
PMNLs: Polymorphonuclear leukocytes
RANKL: Receptor activator of nuclear factor kappa-B ligand
TADs: Temporary anchorage devices
TNF-α: Tumour necrosis factor alpha

## References

[1] Migliorati M, Drago S, Gallo F, Amorfini L, Dalessandri D, Calzolari C, Benedicenti S, Silvestrini-Biavati A. Immediate versus delayed loading: comparison of primary stability loss after miniscrew placement in orthodontic patients-a single-centre blinded randomized clinical trial. Eur J Orthod. 2016;38(6):652–59. 10.1093/ejo/cjv095 26728036

[2] Shank SB, Beck FM, D’Atri AM, Huja SS (2012). Bone damage associated with orthodontic placement of miniscrew implants in an animal model. Am J Orthod Dentofac Orthop.

[3] Chen Y, Kyung HM, Zhao WT, Yu WJ (2009). Critical factors for the success of orthodontic mini-implants: a systematic review. Am J Orthod Dentofac Orthop.

[4] Freitas AOA, Alviano CS, Alviano DS, Siqueira JF, Nojima LI, Nojima Mda CG (2012). Microbial colonization in orthodontic mini-implants. Braz Dent J.

[5] Monga N, Chaurasia S, Kharbanda OP, Duggal R, Rajeswari MR (2014). A study of interleukin 1beta levels in peri-miniscrew crevicular fluid (PMCF). Prog Orthod.

[6] Kapoor P, Kharbanda OP, Monga N, Miglani R, Kapila S (2014). Effect of orthodontic forces on cytokine and receptor levels in gingival crevicular fluid: a systematic review. Prog Orthod.

[7] Nagamatsu J. Bone response to orthodontic miniscrew placement: an in vivo study [Unpublished Master’s Thesis]. Saint Louis University; 2008 [cited 2016 Feb 23]. Available from: https://www.slu.edu/Documents/cade/thesis/Nagamatsu_Thesis.pdf

[8] Bartold PM, Kuliwaba JS, Lee V, Shah S, Marino V, Fazzalari NL (2011). Influence of surface roughness and shape on microdamage of the osseous surface adjacent to titanium dental implants. Clin Oral Implants Res.

[9] Eriksson AR, Albrektsson T (2017). Temperature threshold levels for heat-induced bone tissue injury: a vital-microscopic study in the rabbit. J Prosthet Dent.

[10] Eriksson RA, Albrektsson T, Magnusson B (1984). Assessment of bone viability after heat trauma. A histological, histochemical and vital microscopic study in the rabbit. Scand J Plast Reconstr Surg.

[11] Kharbanda OP, Kapoor PMR, Kapila SD, KWL V, Huang GJE (2017). Peri-miniscrew biomarkers as indicators for miniscrew stability or failure. Anecdote, expertise and evidence: applying new knowledge to everyday orthodontics.

[12] El-Omar EM, Carrington M, Wong-Ho C, McColl KEL (2000). Interleukin-1 polymorphisms associated with increased risk of gastric cancer. Nature.

[13] Gowen M, Wood DD, Ihrie EJ, McGuire MK, Russell RG (1983). An interleukin 1 like factor stimulates bone resorption in vitro. Nature.

[14] Kanneganti T-D (2010). Central roles of NLRs and inflammasomes in viral infection. Nat Rev Immunol.

[15] Yi-June L, Cheing-Meei L, Man-Ying W, Lein-Tuan H, Wei-Kuei C (1999). Interleukin 1β-secreting cells in inflamed gingival tissue of adult periodontitis patients. Cytokine.

[16] Kao RT, Curtis DA, Richards DW, Preble J (1995). Increased interleukin-1 beta in the crevicular fluid of diseased implants. Int J Oral Maxillofac Implants.

[17] Sari E, Uçar C (2007). Interleukin 1beta levels around microscrew implants during orthodontic tooth movement. Angle Orthod..

[18] Baggiolini M, Walz A, Kunkel SL (1989). Neutrophil-activating peptide-1/interleukin 8, a novel cytokine that activates neutrophils. J Clin Invest.

[19] Wilson M, Reddi K, Henderson B (1996). Cytokine-inducing components of periodontopathogenic bacteria. J Periodontal Res.

[20] Scarel-Caminaga RM, Trevilatto PC, Souza AP, Brito RB, Line SRP (2002). Investigation of an IL-2 polymorphism in patients with different levels of chronic periodontitis. J Clin Periodontol.

[21] Dienz O, Rincon M (2009). The effects of IL-6 on CD4 T cell responses. Clin Immunol.

[22] Ishimi Y, Miyaura C, Jin CH, Akatsu T, Abe E, Nakamura Y (1990). IL-6 is produced by osteoblasts and induces bone resorption. J Immunol.

[23] Bartold PM, Haynes DR (1991). Interleukin-6 production by human gingival fibroblasts. J Periodontal Res.

[24] Harada A, Sekido N, Akahoshi T, Wada T, Mukaida N, Matsushima K (1994). Essential involvement of interleukin-8 (IL-8) in acute inflammation. J Leukoc Biol.

[25] Venza I, Visalli M, Cucinotta M, De Grazia G, Teti D, Venza M (2010). Proinflammatory gene expression at chronic periodontitis and peri-implantitis sites in patients with or without type 2 diabetes. J Periodontol.

[26] Tuncer BB, Özmeriç N, Tuncer C, Teoman I, Çakilci B, Yücel A (2005). Levels of interleukin-8 during tooth movement. Angle Orthod.

[27] Hamamci N, Acun Kaya F, Uysal E, Yokucs B (2011). Identification of interleukin 2, 6, and 8 levels around miniscrews during orthodontic tooth movement. Eur J Orthod.

[28] Kaya FA, Hamamci N, Uysal E, Yokuş B (2011). Identification of tumor necrosis factor-α levels around miniscrews during canine distalization. Korean J Orthod.

[29] Lowney JJ, Norton LA, Shafer DM, Rossomando EF (1995). Orthodontic forces increase tumor necrosis factor α in the human gingival sulcus. Am J Orthod Dentofac Orthop.

[30] Schierano G, Pejrone G, Brusco P, Trombetta A, Martinasso G, Preti G (2008). TNF-α, TGF-β2 and IL-1β levels in gingival and peri-implant crevicular fluid before and after de novo plaque accumulation. J Clin Periodontol.

[31] Kuroda Y, Maruyama K, Fujii H, Sugawara I, Ko SBH, Yasuda H (2016). Osteoprotegerin regulates pancreatic β-cell homeostasis upon microbial invasion. PLoS One.

[32] Ogasawara T, Yoshimine Y, Kiyoshima T, Kobayashi I, Matsuo K, Akamine A (2004). In situ expression of RANKL, RANK, osteoprotegerin and cytokines in osteoclasts of rat periodontal tissue. J Periodontal Res.

[33] Van Kooten G, Banchereau J (1996). CD40-CD40 ligand: a multifunctional receptor-ligand pair. Adv Immunol.

[34] Enhos S, Veli I, Cakmak O, Ucar FI, Alkan A, Uysal T (2013). OPG and RANKL levels around miniscrew implants during orthodontic tooth movement. Am J Orthod Dentofac Orthop.

[35] Simanek V, Kren V, Ulrichová J, Gallo J (2005). The efficacy of glucosamine and chondroitin sulfate in the treatment of osteoarthritis: are these saccharides drugs or nutraceuticals. Biomed Pap Med Fac Univ Palacky Olomouc Czech Repub.

[36] Waddington RJ, Embery G, Samuels RHA (1994). Characterization of proteoglycan metabolites in human gingival crevicular fluid during orthodontic tooth movement. Arch Oral Biol.

[37] Smedberg J-I, Beck CB, Embery G (1993). Glycosaminoglycans in peri-implant sulcus fluid from implants supporting fixed or removable prostheses. Clin Oral Implants Res.

[38] Last KS, Smith S, Pender N (1995). Monitoring of IMZ titanium endosseous dental implants by glycosaminoglycan analysis of peri-implant sulcus fluid. Int J Oral Maxillofac Implants.

[39] Johansson B, Smedberg J-I, Langley M, Embery G (2001). Glycosaminoglycans in peri-implant sulcus fluid from implants placed in sinus-inlay bone grafts. Clin Oral Implants Res.

[40] Intachai I, Krisanaprakornkit S, Kongtawelert P, Ong-Chai S, Buranastidporn B, Suzuki EY (2010). Chondroitin sulphate (WF6 epitope) levels in peri-miniscrew implant crevicular fluid during orthodontic loading. Eur J Orthod.

[41] Billingham MEJ. Cytokines as inflammatory mediators. Br Med Bull. 1987;43(2):350–70. 10.1093/oxfordjournals.bmb.a072187 3319033

[42] Alhashimi N, Frithiof L, Brudvik P, Bakhiet M (2001). Orthodontic tooth movement and de novo synthesis of proinflammatory cytokines. Am J Orthod Dentofac Orthop.

[43] Jäger A, Zhang D, Kawarizadeh A, Tolba R, Braumann B, Lossdörfer S, et al. Soluble cytokine receptor treatment in experimental orthodontic tooth movement in the rat. Eur J Orthod. 2005;27(1):1–11. 10.1093/ejo/cjh089 15743857

[44] Kanzaki H, Chiba M, Arai K, Takahashi I, Haruyama N, Nishimura M (2006). Local RANKL gene transfer to the periodontal tissue accelerates orthodontic tooth movement. Gene Ther.

[45] Kanzaki H, Chiba M, Shimizu Y, Mitani H (2001). Dual regulation of osteoclast differentiation by periodontal ligament cells through RANKL stimulation and OPG inhibition. J Dent Res.

[46] Waddington RJ, Embery G, Last KS (2017). Glycosaminoglycans of human alveolar bone. Arch Oral Biol.

